# Cross-Modal Transfer Following Auditory Task-Switching Training in Old Adults

**DOI:** 10.3389/fpsyg.2021.615518

**Published:** 2021-02-25

**Authors:** Benjamin Robert William Toovey, Florian Kattner, Torsten Schubert

**Affiliations:** ^1^Department of Psychology, Martin-Luther University Halle-Wittenberg, Halle, Germany; ^2^Institute of Psychology, Technische Universität Darmstadt, Darmstadt, Germany

**Keywords:** task-switching training, cross-modal transfer, executive functions, cognitive plasticity, cognitive aging

## Abstract

Maintaining and coordinating multiple task-sets is difficult and leads to costs, however task-switching training can reduce these deficits. A recent study in young adults demonstrated that this training effect occurs at an amodal processing level. Old age is associated with reduced cognitive plasticity and further increases the performance costs when mixing multiple tasks. Thus, cognitive aging might be a limiting factor for inducing cross-modal training effects in a task-switching environment. We trained participants, aged 62–83 years, with an auditory task-switching paradigm over four sessions (2880 total trials), to investigate whether training-related reductions in task-switching costs would also manifest in an untrained visual modality version of the task. Two control groups trained with single tasks (active control) or not trained (passive control) allowed us to identify improvements specific to task-switching training. To make statistical evaluations of any age differences in training and cross-modal transfer, the data from the Kattner cohort were incorporated into the present analysis. Despite the tendency for older adults to respond more cautiously, task-switching training specifically led to a mixing cost reduction in both trained and untrained modalities, the magnitude of which was statistically similar regardless of age. In line with a growing body of research, we failed to observe any far transfer effects in measures of inhibition, working memory or fluid intelligence. Overall, we conclude that any apparent cognitive limitations associated with aging do not prevent cognitive control processes which support set-shifting from improving at an amodal level.

## Cross-Modal Transfer Following Auditory Task-Switching Training in Old Adults

Flexible adaptation of cognition and action is demanding. This flexibility is dependent on executive control. Although healthy aging (Ridderinkhof et al., 2002) or neurological damage (Rogers et al., 1998; Shallice et al., 2008) may impede the efficient adaptation to environmental demands, improvements in executive functions have been reported following working memory (e.g., Jaeggi et al., 2008), dual-tasks (e.g., Schubert et al., 2017) and even video game training (e.g., Strobach et al., 2012a), suggesting generalized cognitive ability is plastic (but see Sala and Gobet, 2019). Psychological research often focuses on set-shifting, which is considered to be an important executive function similar to updating, inhibition and dual-tasking (Miyake et al., 2000; Diamond, 2013). Task-switching paradigms tap into several of these executive functions, most prominently set-shifting (Strobach et al., 2014), and a large body of research indicates that performance in task-switching situations can also be improved (Rogers and Monsell, 1995; Kray and Lindenberger, 2000; Stoet and Snyder, 2007; Minear and Shah, 2008; Karbach and Kray, 2009; Strobach et al., 2012b; Pereg et al., 2013; Kattner et al., 2019).

The mechanisms behind such improvements, however, are yet unclear. One possibility is that individuals develop task-specific strategies, such as mnemonic or imagery techniques, producing improvements which are restricted to the trained task, but also to structurally similar but untrained tasks (“near transfer”). Another possibility is that the executive functions supporting task-switching themselves are trained. An interesting consequence of this hypothesis is that beneficial effects will also occur in structurally unrelated tasks that share underlying executive functions with the trained task (“far transfer”). Indeed, such process-based training effects have been reported in task-switching paradigms (Karbach and Kray, 2009). Recently, evidence for a further indicator of process-based improvement following task-switching training was provided in the form of cross-modal transfer suggesting that the mechanisms behind task set-shifting are amodal, and training effects may not simply reflect improved attention to set-shifts of stimuli in a single trained modality. In this paper, we seek to develop upon this new cross-modal training effect as an indicator for process-based training, by exploring whether it relates to healthy aging.

In a typical task-switching paradigm (Jersild, 1927; Rogers and Monsell, 1995), participants process task-relevant visual stimuli based on an instructed task set (auditory stimuli are rarely used; Seibold et al., 2018). Trials for two or more tasks can be presented in separate blocks or alternated together in mixed-task blocks wherein both switching and repetition of task sets can occur. In mixed-task blocks, mean performance differences for switch and repeat trials indicate the transient cognitive demand of set shifting (switch cost). Mean performance differences between repeated trials in mixed and single-task blocks indicate the sustained cognitive demand associated with coordinating and maintaining concurrent tasks (mixing costs; Rogers and Monsell, 1995; Monsell, 2003). Importantly for the present work, these two costs have been dissociated by both aging, as well as training and transfer effects.

### Aging Effects

Some costs associated with task-switching are increased as a function of healthy aging. The corresponding literature in task-switching contends that (1) mixing cost magnitudes increase with increasing age (Reimers and Maylor, 2005) and (2) switch cost magnitudes do not vary substantially as a function of aging (Kray and Lindenberger, 2000; Reimers and Maylor, 2005; for a meta-analytical approach see Wasylyshyn et al., 2011). Despite visual stimuli being used predominantly in task-switching paradigms, tasks with auditory stimuli additionally indicate that while older adults are generally slower to respond, switch costs did not differ between young and older adults (Lawo and Koch, 2014; Getzmann et al., 2017). However, it must be noted that under specific circumstances, such as set-shift unpredictability, switch costs may increase with aging (Cepeda et al., 2001; Kray et al., 2002).

Older adults' cognitive flexibility is reduced, and this is likely reflected in their larger mixing cost magnitudes relative to more youthful peers (Lövdén et al., 2010). Meanwhile, the processes related to implementing the individual set-shifts on each trial must be relatively well-preserved, if we are to interpret the stability of the switch cost as reflecting these. The dissociation of the two costs as a function of aging indicates clear differences in the stability of the cognitive mechanisms which support each effect. However, baseline differences in flexibility between young and old adults may be a limiting factor for general improvements in either of these two task-switching related costs, or the translation of improvements to other tasks, as we discuss in the following section.

### Training and Transfer Effects

Task-switching performance and its associated costs, and therefore presumably the underlying processes, can be improved with training. While training reduces both types of cost, mixing costs can be completely eliminated (Berryhill and Hughes, 2009; Strobach et al., 2012b; but see Zhao et al., 2018), but residual switching costs persist, even after hundreds of thousands of trials (Berryhill and Hughes, 2009; Salminen et al., 2012; see also Stoet and Snyder, 2007; Zhao et al., 2018). Importantly, the training and aging factors appear to interact at the level of mixing costs. Specifically, older adults have exhibited larger training related reductions following task-switching training (Kray and Lindenberger, 2000; Buchler et al., 2008; Karbach and Kray, 2009), sometimes reducing to match the performance of young adults (Gaál and Czigler, 2018; Steyvers et al., 2019).

Cognitive training with task-switching may therefore be valuable to aging individuals as a means of increasing their cognitive flexibility (Lövdén et al., 2010), and in the case of task switching this may be particularly beneficial for the executive control processes more explicitly linked to the mixing cost, such as working memory, inhibition and competition control (Rubin and Meiran, 2005). If such training leads to improvements in the underlying processes that are shared with other tasks, then the value of such cognitive training is significantly increased, since training in one task can transfer its benefits to others (Taatgen, 2013; Schubert et al., 2014; Strobach et al., 2014). Indeed, a recent study demonstrated convincingly that tasks measuring inhibition and fluid intelligence were improved following task-switching training, indicating the presence of far transfer (Karbach and Kray, 2009). However, several further studies employing a similar design have since failed to replicate the key findings of far transfer (Pereg et al., 2013; Kray and Fehér, 2017; Zhao et al., 2018; Kattner et al., 2019), although near transfer effects appear to be more replicable. Therefore, the reliability of observing far transfer to structurally dissimilar tasks following task-switching training remains to be seen. One further indicator for the occurrence of transfer effects following task-switching training is the case of cross-modal transfer. While Karbach and Kray (2009) demonstrated transfer effects following task-switching training, these were modality-*dependent* effects; despite the training and task stimulus sets differing, they were both presented in the visual modality (see Yeung et al., 2006). In contrast, the modality-*independent* nature of executive functions (i.e., where the modality of the testing and training stimuli are alternated) supporting task-switching remains poorly explored (Koch et al., 2018). Indeed, neuroscientific evidence seems to point toward modality-independent activity in the prefrontal cortex, recorded during executive function tasks (Tamber-Rosenau et al., 2013), thus providing biological grounds for such assumptions in task-switching.

Alternatively, training effects in task-switching could be dependent the trained modality. Smaller task-switching costs were shown when modality alternated from visual to auditory stimuli between trials (or vice versa), than when the modality repeated (Murray et al., 2009). This suggests that stimulus-modality specific neural pathways are separable from each other in a task-switching situation, and thus task-switching training may eventually be restricted to the modality in which they were trained, rather than improving processes an amodal level (Pashler and Baylis, 1991; Schubert et al., 2014).

To address this open question, Kattner et al. (2019) investigated whether training of an auditory task-switching situation may lead to cross-modal training and transfer effects to an untrained visual task version in young adults. Four auditory task-switching training sessions were sandwiched between two assessment sessions. In the pre and post-test sessions, a visual modality version of the auditory training task was administered, enabling the authors to assess cross-modal transfer. The remaining assessments tested for far transfer in different executive function tasks, like those investigated in Karbach and Kray (2009). Two results of critical importance were observed. First, auditory-modality task-switching training led to reductions in the size of the auditory-modality mixing cost. Second, exclusive to this training group, but not for either of the two control groups, mixing costs in the untrained visual modality version of the task were also reduced in size. These results demonstrated that cognitive training effects indexed by the mixing cost occurred at an amodal level of task processing. Finally, in accordance with several studies which followed Karbach and Kray (2009)'s original paper, Kattner et al. failed to observe any far transfer effects (Pereg et al., 2013; Kray and Fehér, 2017; Zhao et al., 2018; Kattner et al., 2019).

### The Present Study

As has been reviewed, task mixing costs are greater in older compared to younger participants, and this may be a result of reduced cognitive flexibility in older individuals (Lövdén et al., 2010). Such reduction in cognitive flexibility is also evident in the tendency for perseverative behaviors in older adults, which has been attributed to deficits in set-shifting ability (Ridderinkhof et al., 2002). One might therefore expect that reduced cognitive flexibility as indexed by the mixing cost in task-switching situations, perhaps as a function of a strategic tendency to persevere on a single task set, would manifest as a reduced ability for amodal learning of set-shifting. Indeed, episodic retrieval accounts of task-switching effects would suggest that the modality of the stimuli is a critical element of the associated task-sets (Waszak et al., 2003; Murray et al., 2009; Lukas et al., 2010), and working memory limitations in older adults may inflate the relevance of the learned modality while restricting flexibility of transfer of learning to alternative modalities. Thus, it is possible that cross-modal transfer effects may be reduced or even absent in this age group at all. However, this assumption is based on studies which assessed single session performance and, thus, fails to consider the training-induced cognitive plasticity reported by task-switching and aging studies (for an overview, see Gajewski et al., 2018). Indeed, it has been observed that mixing cost reductions following task-switching training appear to be even greater in older compared with younger adults (Gaál and Czigler, 2018; Steyvers et al., 2019), which conversely indicate a potential for superior optimization with training.

Integrating the literature reviewed thus far, we intend to expand upon the recent findings of cross-modal transfer following training in auditory switching tasks in young adults, by examining if this amodal training effect is preserved in older adults. We will first establish whether older adults can benefit from auditory task-switching training, since to our knowledge there are no experimental task switching studies in the elderly populations which employ auditory-modality training paradigms. Secondly, and crucially, we aim to test whether cross-modal transfer effects occur in these individuals, since it is presently unclear whether such amodal processing ability would be limited by age. Thus, we applied the exact design of Kattner et al. (2019) to a group of old adults aged 60 and older, in order to assess training, and near or far-transfer effects in an auditory task-switching paradigm. To dissect task-switching related benefits from non-specific advantages of training, i.e., ones that may occur due to improvements in each component task, an active training group were training on the task-switching protocol where trials from two auditory categorization tasks were alternated. An active control group were also trained, but the two tasks were presented independently of one another, thus, leaving out the necessity to switch between the two tasks. Finally, the passive control group was not trained at all (see also Strobach et al., 2012b; Schubert et al., 2017; for discussions of the value of this approach). According to results already obtained in the visual domain comparing older and younger participants (Karbach and Kray, 2009), we expected to see significant training-related reductions in the auditory domain mixing costs of the older participants. Secondly, we expected to see, like the previous experiment on younger individuals (Kattner et al., 2019), cross-modal transfer effects from the trained auditory to the untrained visual domain in older adults. Indeed, related literature in dual-task studies is suggestive of preserved amodal processing capacity in healthy aging (Lussier et al., 2012). Finally, as a supplementary step, we obtained with permission the data from the Kattner et al. (2019) cohort, and conducted secondary analyses comparing the training-related performance of older and younger subject groups. We reasoned that this would allow us to make statistically justified inferences about any age-related differences we observe in training and cross-modal transfer effects.

Provided we observe auditory training improvements which also manifest in the cross-modal scenario, then in a narrow perspective this pattern of results would be a first documentation of training effects following task-switching training in the auditory modality in older participants. In addition, significant cross-modal transfer effects to the untrained visual task switching situation for the older participants would be consistent with a view that executive function capabilities are preserved throughout aging. Furthermore, this would demonstrate that the potential for executive control skills to be trained at an amodal level exists even for older participants. This would further bolster the evidence that cross-modal transfer provides a reliable alternative marker for process-based training effects in task-switching designs, besides far-transfer.

Finally, we included several additional task situations drawing on inhibitory control, working memory distraction and fluid intelligence to assess whether there are signs for far transfer effects after auditory task-switching in older subjects. For instance, the absence of far transfer in younger subjects reported by Kattner et al. (2019) does not speak to the possibility of far transfer effects in an older cohort with this auditory attention shifting design, as such transfer effects in the visual modality were observed in older participants by Karbach and Kray (2009).

## Methods

### Participants

Thirty-seven participants, representing both senior colleagues of the university and citizens were recruited via opportunity sampling using flyers in the city of Halle (Saale), Germany. One participant did not finish due to mitigating circumstances. Thirty-six participants (19 females, 62–83 years, *M*_Age_ = 69.9 years, *SD*_Age_ = 5.7 years) were randomly and evenly assigned to one of three groups (*n* = 12 per group). These were the *Active Training* (AT) group (task-switch training: 5 females, 62–78 years, *M*_Age_ = 68.9 years, *SD*_Age_ = 5.1 years), the *Active Control* (AC) group (single-task training: 6 females, 65–83 years, *M*_Age_ = 70.3 years, *SD*_Age_ = 5.4 years) and the *Passive Control* (PC) group (no training: 8 females, 62–81 years, *M*_Age_ = 70.6 years, *SD*_Age_ = 6.8 years). All participants reported normal or corrected-to-normal hearing and vision, provided written informed consent and were reimbursed with ~€8.80 per hour of their time. Since an a priori power analysis was not conducted, a sensitivity analysis was instead conducted using G*Power 3.1.9.7 (Faul et al., 2007). Optimized around the detection of a significant interaction between training group (between group with three levels) and testing point (within factors with two levels) of the auditory modality mixing cost, the sample size of this study had an 80% power to detect a minimum reliable effect size of 0.27 (cf. 0.25 medium effect size normally used for a priori sample-size calculations).

The research protocol detailed herein received local ethical approval from Technical University of Darmstadt for Kattner et al. (2019).

To control for possible between-group differences in baseline performance which may confound interpretations of training-related improvements, participants were semi-randomly assigned to one of the three experimental groups via titration according to Green et al. (2019): after random assignment of nine participants into each of the groups, group summary statistics for reaction time in the pre-test auditory and visual single and switching blocks were calculated. Then for each new participant, individual single task performance was calculated, and the following participants were assigned such that the difference between the summary statistics of the three groups remained smallest.

The primary interest of the present study was to investigate whether older adults trained with task-switching would exhibit auditory-to-visual cross-modal transfer in a range of near and far transfer assessments. Therefore, all analyses are presented first based on the data of these participants. In a secondary step, each analysis is supplemented with a cross-experimental ANOVA, where the data from the younger cohort of Kattner et al. (2019) are included as an additional between-groups factor. The young cohort of participants was recruited at the campus of the Technical University of Darmstadt, Germany. Participants (*n* = 57) were randomly assigned to one of the three experimental groups, which were one AT group (11 females, 19–27 years, *M*_Age_ = 21.5 years), one AC group (11 females, 19–31 years, *M*_Age_ = 22.1 years) and one PC group (10 females, 19–58 years, *M*_Age_ = 28.8 years), with *n* = 19 in each group.

### Apparatus

The experiment took place in one of two single-walled sound-attenuated booths. All instructions and stimuli for tasks in the visual modality were presented on a 24-inch Viewsonic XG2401 monitor. Auditory stimuli were presented dichotically through Sennheiser HD 471 headphones. The experimental routines were programmed in MATLAB (Mathworks, Natick, MA, USA) utilizing the Psychophysics toolbox extensions (Brainard, 1997; Pelli, 1997; Kleiner et al., 2007), and presented using a desktop computer running Microsoft Windows 10.

### Stimuli

For the auditory switching tasks in both pre- and post-tests and all training scenarios, speech recordings of a female-voice vocalizing eight German numerals from two to nine (“zwei” to “neun”), to be categorized as odd or even, and eight German plural nouns of fruits (“Birnen,” “Kirschen,” “Melonen,” “Zitronen”) and vegetables (“Zwiebeln,” “Bohnen,” “Gurken,” “Tomaten”), to be categorized in their respective types, were used. These stimulus sets formed the two component tasks, and were presented dichotically, with one word being presented to the left ear and the other word being presented to the right ear. The ear to which the words from each task set were presented was randomly assigned on each trial.

### Procedure

The multi-day experiment consisted of a pre-test session on the first day, a post-test session on the last day, and four intermediate training sessions for the AT and the AC groups. Training sessions were scheduled within a range of 4–48-h intervals, limited to two sessions per day. The PC group completed only the pre- and the post-test sessions, which were separated by about 2 weeks.

### Pre- and Post-Tests

Six different cognitive tests were administered in a counterbalanced order (Latin-square method); (1) auditory-modality task-switching with the two aforementioned tasks, (2) visual-modality task-switching, (3) Number Stroop (measuring response inhibition), (4) Digit Span (measuring verbal memory interference), (5) Corsi Span (measuring spatial memory interference), and (6) Raven's Advanced Progressive Matrices (measuring fluid intelligence). Tests 1 and 2 comprised the near, cross-modal transfer and training assessments, and tests 3–6 comprised the far-transfer assessments. These sessions lasted ~60–90 min.

*Auditory task switching* began with two 24 trial single-task blocks followed by a mixed-task block of 48 trials. Each block was preceded by eight practice trials, for which the data were not analyzed. The fruit/vegetable categorization block occurred first, followed by the odd/even categorization block and finally the mixed block, in which task switches occurred every second trial (alternating runs, with run length *n* = 2; Rogers and Monsell, 1995). All trials began with a central fixation cross for 750 ms. Bivalent stimulus presentation was achieved by presenting a randomly drawn food word to one ear, and a randomly drawn number word to the other simultaneously, with stimulus-ear allocation randomized every trial. A light gray text cue was presented on screen at the same time as the auditory stimulus array, reminding participants of the current task (“Obst – Gemüse” or “Ungerade – Gerade”). Participants were required to respond as fast and accurately as possible by pressing the left arrow key on a standard keyboard for fruit words or odd numbers, or the right arrow key for vegetable words or even numbers. Visual feedback was provided for correct, correct-but-slow (1,000 ms < RT < 5,000 ms), and incorrect or absent (RT > 5,000 ms) responses. A new trial started after the feedback message.

*Visual task switching* was identical to the auditory task-switching procedure except that the numerals and nouns were presented visually as text on the monitor. The two words (one from each task) were randomly positioned centrally to the left or right of fixation.

The *Number Stroop* task required participants to identify the number of characters presented on screen in each trial by pressing the respective number on the keyboard. In neutral trials, characters were capital letters “H,” “K,” “L,” “P,” and in congruent or incongruent trials they were the numbers 1-4. Between one and four characters could be presented on screen in a single trial. A total of 192 trials were presented in a random order, representing 16 repetitions of all possible trial type x stimulus combinations, i.e., 1/3 compatible, 1/3 neutral, and 1/3 incompatible trials. An additional practice block containing one of each possible combination (i.e., 12 trials) was presented before the block, but not analyzed. Trials began with a 500 ms fixation, and stimuli were presented for 2,000 ms or until participant response, whichever came first.

In the *Digit Span* task, a random sequence of eight digits (from 1 to 9) were presented on each trial. In half of all trials, digits were presented visually on screen for 1,000 ms each, and in the other half they were presented dichotically via headphones. After 6,000 ms retention, participants clicked on a 3 × 3 numeric pad, presented on screen, the identity of the eight digits in serial order. Visual performance feedback was presented for 1,000 ms following the eighth digit. Phonological interference with verbal memory was measured with 14 s of either free-running Finnish speech or white noise presented as a task-irrelevant sound, during the presentation of digits and the retention interval. Each trial type was repeated five times for a total of 20 trials. Two additional practice trials were presented before the main task which were not analyzed.

The *Corsi Span* task involved 24 trials of digitally presented, sequential Corsi blocks. Six randomly chosen target blocks from an array of 16 empty blocks, presented on screen, were successively highlighted by filling them with red color for 1,250 ms each. Following 5,000 ms retention, the 16 empty blocks were presented once more, and participants were asked to click on screen the location of the target blocks in serial order. Interference with spatial memory was measured by introducing additional irrelevant colored blocks every 1,250 ms on half of the trials, presented at random locations in the gaps between empty squares, during both presentation of target blocks and the retention interval. Text feedback about performance was provided before the next trial started.

Finally, *fluid intelligence* was measured by administering the 36-item short form of Raven's Advanced Progressive Matrices test (Arthur and Day, 1994; John, 2003). At pre-test the 18 odd-numbered items were presented and the remaining items at post-test. Participants had 10 min for all 18 problems, but no response deadline was given for any individual problem. Previous responses were not allowed to be changed, and feedback was not presented.

### Training Tasks

The training groups were trained with both task-sets in all sessions, for a total of 2,880 trials, comprised of 15 blocks of 48 trials, for 720 trials per session. Each training session lasted approximately about 30–40 min, and after each block participants could take a short break. In the AT group, the two tasks were mixed within blocks such that tasks alternated systematically every other trial (task order AABBAABB…). In the AC group, the two component tasks were presented separately, alternating blockwise. Trial structure, including feedback provision, was identical to that observed by participants in the pre- and post-test versions. The PC group did not get any training in the time interval between the pre- and post-test sessions.

## Results

### Data Report

To assess whether older participants are capable of training-related improvements in auditory task switching, we report the training data, and the data of the training-related changes in the auditory mixing and switching costs in this group. Afterwards, we report the transfer effects in old participants. For that purpose, we, first report cross-modal transfer effects of the auditory task switching training to the mixing and switching costs in the visual task switching situations, and then report the far transfer effects of auditory task switching training on performance in unrelated tasks. Finally, we compare the training- and transfer-effects in the older cohort to the corresponding effects obtained in a control group of younger participants, who were exposed to the same paradigms in an earlier study of Kattner et al. (2019). Tables 1, 2 presents descriptive statistics for the pre and post-test data in the old and young age groups, respectively.

**Table 1:** Mean RTs (ms) in the task switching assessments at pre- and post-test, for the average of the two single task blocks with repeat trials, the average of repeat trials in the mixed block, and the average of the switch trials in the mixed block in old adults.

**Trial type**		**Test**	**PC group**	**AC group**	**AT group**
Single block (repeat)	Visual	Pre	915 (153)	933 (262)	821 (111)
		Post	891 (208)	777 (104)	818 (129)
	Auditory	Pre	809 (229)	755 (283)	662 (241)
		Post	666 (179)	414 (172)	530 (150)
Mixed block (repeat)	Visual	Pre	1,654 (479)	1,552 (497)	1,548 (287)
		Post	1,674 (458)	1,364 (364)	1,223 (157)
	Auditory	Pre	1,446 (360)	1,337 (371)	1,348 (321)
		Post	1,359 (288)	1,054 (435)	772 (105)
Mixed block (switch)	Visual	Pre	1,662 (460)	1,518 (479)	1,573 (370)
		Post	1,608 (460)	1,367 (415)	1,220 (181)
	Auditory	Pre	1,491 (375)	1,382 (353)	1,363 (314)
		Post	1,377 (298)	1,121 (491)	759 (88)

*Standard deviations are shown in parentheses*.

**Table 2:** Mean RTs (ms) in the task switching assessments at pre- and post-test, for the average of the two single task blocks with repeat trials, the average of repeat trials in the mixed block, and the average of the switch trials in the mixed block in young adults (Data are based on the study of Kattner et al., 2019).

**Trial type**		**Test**	**PC group**	**AC group**	**AT group**
Single block (repeat)	Visual	Pre	767 (124)	729 (127)	726 (83)
		Post	718 (101)	626 (72)	632 (71)
	Auditory	Pre	675 (99)	664 (131)	629 (129)
		Post	599 (123)	423 (64)	468 (129)
Mixed block (repeat)	Visual	Pre	1,378 (269)	1,241 (215)	1,256 (249)
		Post	1,164 (200)	1,029 (178)	893 (214)
	Auditory	Pre	1,119 (155)	1,112 (178)	1,068 (149)
		Post	1,001 (150)	839 (124)	617 (177)
Mixed block (switch)	Visual	Pre	1,398 (248)	1,223 (199)	1,310 (240)
		Post	1,180 (232)	1,056 (218)	934 (218)
	Auditory	Pre	1,179 (182)	1,120 (156)	1,103 (191)
		Post	1,028 (182)	930 (135)	659 (188)

*Standard deviations are shown in parentheses*.

### Training of Auditory Task Switching

**Figure 1:**
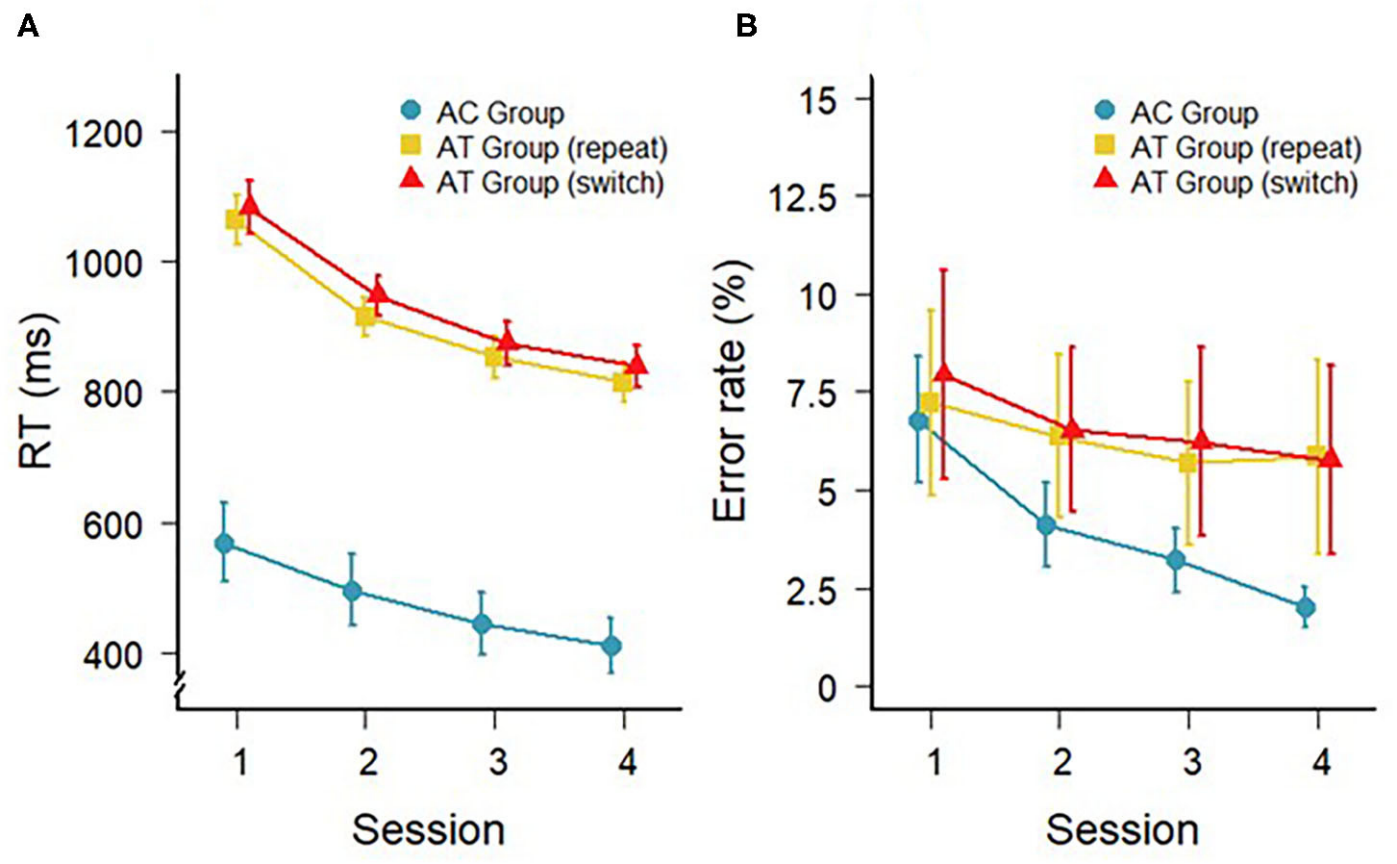
**(A)** Mean RTs (in ms) and **(B)** mean error rates on repeat and switch trials during task-switching training of the AT group and during the single task training of the AC group (repeat trials only). Error bars depict standard errors of the mean.

The response times (RTs) during the four sessions of training with the auditory task for the AT and AC groups are shown in Figure 1A. In the AT group, a 4 (session) × 2 (trial type: switch, repeat) repeated-measures ANOVA confirmed the general decrease in RTs with a main effect of session, *F*_(3,33)_ = 75.60; *p* < 0.001; $\eta_{\text{G}}^{2}$ = 0.41, as well as a marginally significant difference between switch and repeat trials, *F*_(1,11)_ = 4.06; *p* = 0.06; $\eta_{\text{G}}^{2}$ = 0.01 (i.e., switch costs). However, there was no interaction between session and trial type, *F*_(3,33)_ = 0.57; *p* = 0.64; $\eta_{\text{G}}^{2}$ < 0.01, indicating that the switch costs did not change over the course of the training. For the AC group, a one-factorial repeated-measures ANOVA on RTs also revealed a significant main effect of training session, *F*_(3,33)_ =28.81; *p* < 0.001; $\eta_{\text{G}}^{2}$ = 0.11, confirming a general decrease in RTs in the single-task training.

The error rates during the different trial types in the AT and AC groups are illustrated in Figure 1B and were analyzed in a similar manner to the RT data. For the AT group, there was a main effect of session indicating a general improvement of accuracy with training, *F*_(3,33)_ = 4.29; *p* = 0.01; $\eta_{\text{G}}^{2}$ = 0.01. However, there was no difference in accuracy between switch and repeat trials, *F*_(1,11)_ = 0.82; *p* = 0.39; $\eta_{\text{G}}^{2}$ < 0.02, and no interaction, *F*_(3,33)_ = 0.58; *p* = 0.63; $\eta_{\text{G}}^{2}$ < 0.01. For the accuracy during training in the AC group, there was also a significant improvement across the four single-task training sessions, *F*_(3,33)_ = 7.48; *p* < 0.001; $\eta_{\text{G}}^{2}$ = 0.19. Please, note that the PC group did not get training and, therefore, there are no training data for this group.

### Data Trimming

Across both modalities and pre and post-test sessions, we included only trials with correct responses (discarding 11.4% of trials) and those with RTs below 3,000 ms (discarding 1.1% of trials).

### Auditory Modality Mixing Costs

**Figure 2:**
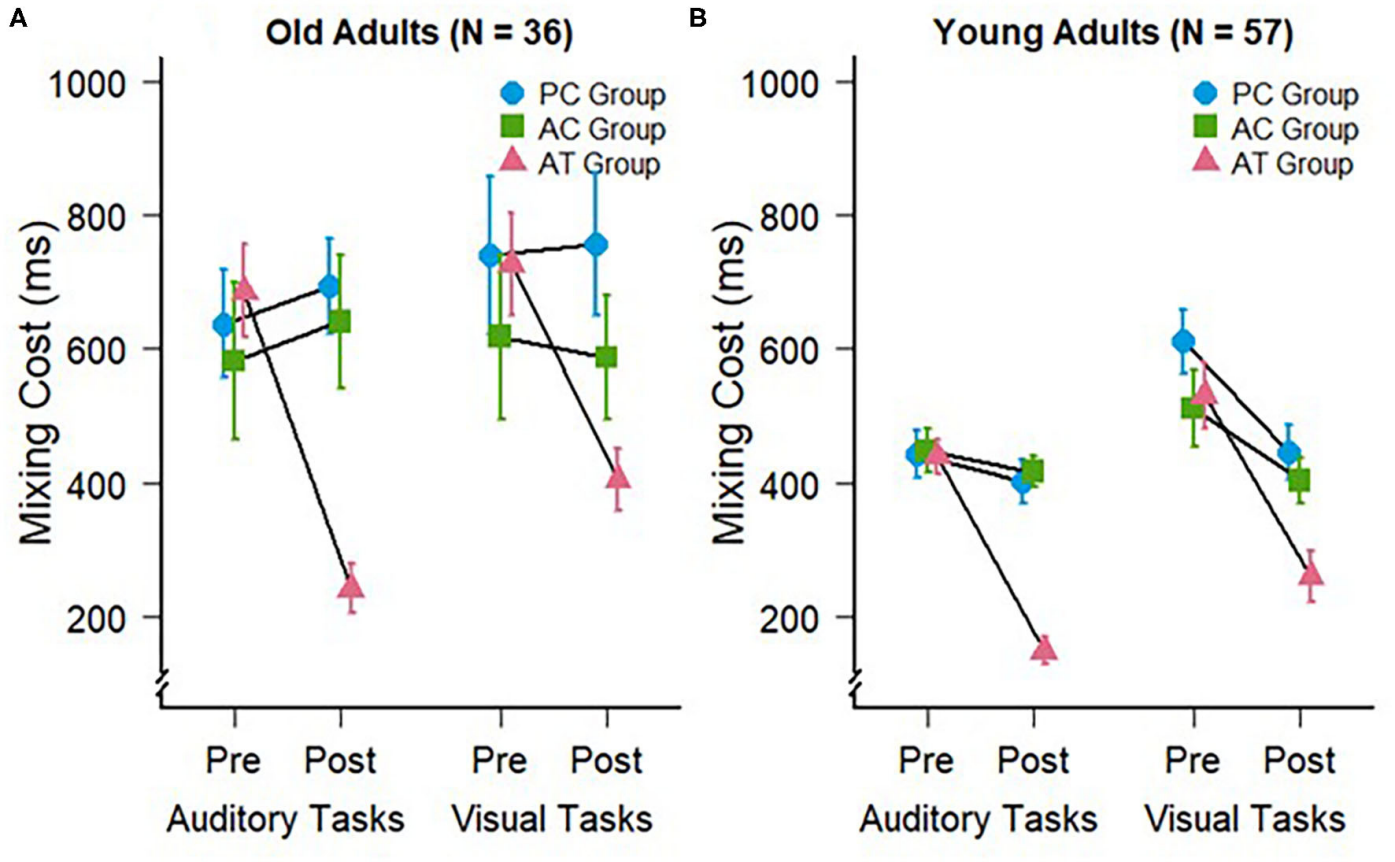
Mixing costs during auditory (trained) and visual (untrained) task switching at pre- and post-test for **(A)** the old adults of the present study and **(B)** young adults of a previous study (Kattner et al., 2019). Error bars depict standard errors of the mean.

To assess whether older participants are capable of improvement in the related executive control functions, we analyzed the mixing costs of the older subjects in auditory at the pre- and post-tests. For that purpose, we subtracted the RTs on single-task blocks from the RTs on repeat trials of mixed-task blocks. The mixing costs at pre- and post-test for the older adults in the present study are depicted in Figure 2A, left panel.

We conducted a 3 (group: AT, AC, PC) × 2 (test: pre vs. post) mixed-factors ANOVA on mixing costs in the auditory modality. This test revealed no significant main effects of group, *F*_(2,33)_ = 2.43; *p* = 0.10; $\eta_{\text{G}}^{2}$ = 0.09, or test, *F*_(1,33)_ = 3.75; *p* = 0.06; $\eta_{\text{G}}^{2}$ = 0.04. However, in accordance with our expectations, there was a significant interaction between group and test, *F*_(2,33)_ = 8.69; *p* < 0.001; $\eta_{\text{G}}^{2}$ = 0.16, confirming the expected specific training effect on auditory mixing costs, as can be seen in Figure 2A. The amount of mixing decreased significantly in the AT group (pre: *M* = 685 ms; *SD* = 238 ms vs. post: *M* = 241 ms; *SD* = 126 ms), *t*_(11)_ = 6.52, *p* < 0.001, but not in the AC (pre: *M* =581 ms; *SD* = 404 ms vs. post: *M* = 640 ms; *SD* = 345 ms), *t*_(11)_ = −0.45, *p* = 0.66, nor the PC group (pre: *M* = 636 ms; *SD* = 279 ms vs. post: *M* = 692 ms; *SD* = 244 ms), *t*_(11)_ = −0.65, *p* = 0.53. These findings indicate for the first time that auditory task switching training can lead to an improvement of auditory mixing costs in older participants, and that the corresponding training-related changes occur only then if an appropriate training requires the permanent processing of the two tasks within a block of trials (AT group). If subjects train the component tasks for the same number of trials but without being required to switch between the two tasks (AC group), then no training-related improvements of switch costs occur.

### Auditory Modality Switching Costs

**Figure 3:**
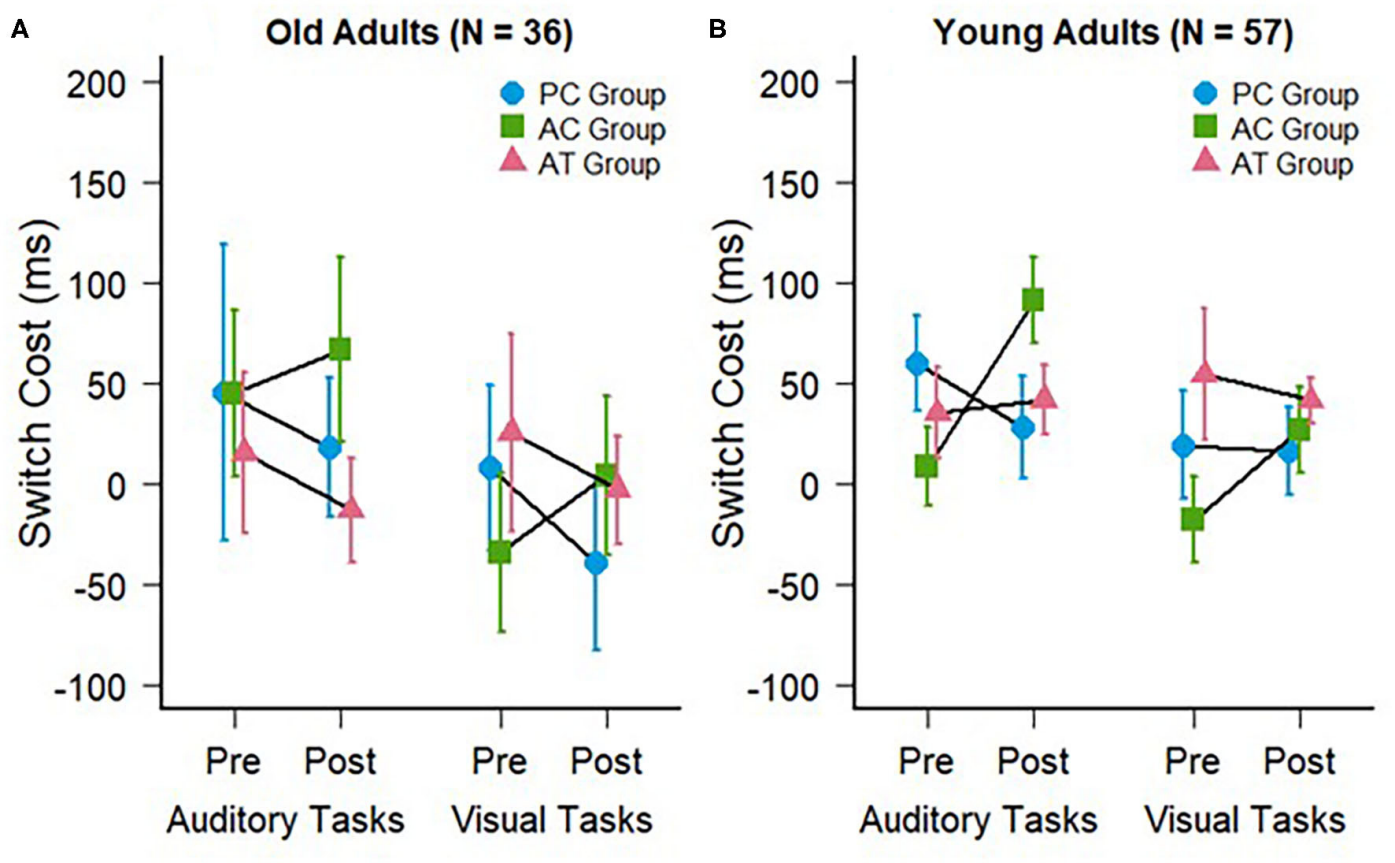
Switch costs during auditory (trained) and visual (untrained) task switching at pre- and post-test for **(A)** the old adults of the present study and **(B)** the young adults of a previous study (Kattner et al., 2019). Error bars depict standard errors of the mean.

The switch costs were calculated by subtracting the RTs on repeat trials from the RTs on switch trials in mixed-task blocks. The auditory and visual switch costs of the old adults in the present study are illustrated in Figure 3A. The mean switch costs were very small in general (AT group pre: *M* = 15 ms; *SD* = 137 ms vs. post: *M* = −13 ms; *SD* = 90 ms; AC group pre: *M* = 45 ms; *SD* = 143 ms vs. post: *M* = 66 ms; *SD* = 158 ms; PC group pre: *M* = 45 ms; *SD* = 254 ms vs. post: *M* = 18 ms; *SD* = 120 ms). A two-way ANOVA for these auditory modality switching costs, with the same factors as the mixing cost above, also revealed no interaction between group and test, *F*_(2,33)_ = 0.23; *p* = 0.79; $\eta_{\text{G}}^{2}$ < 0.01, confirming the absence of a training-related reduction of auditory switch costs in the AT group. There was also no main effect of group, *F*_(2,33)_ = 0.61; *p* = 0.55; $\eta_{\text{G}}^{2}$ = 0.02, or test, *F*_(1,33)_ = 0.11; *p* = 0.74; $\eta_{\text{G}}^{2}$ < 0.01.

### Cross-Modal Transfer: Visual Modality Mixing Costs

To address our hypothesis that older adults would also exhibit training-specific cross-modal transfer to the visual modality, we conducted an ANOVA for the visual task data with the same factors as for the auditory task-switching situation. This ANOVA revealed a significant main effect of test, *F*_(1,33)_ = 4.32; *p* = 0.045; $\eta_{\text{G}}^{2}$ = 0.03, but no main effect of group, *F*_(2,33)_ = 1.27; *p* = 0.29; $\eta_{\text{G}}^{2}$ = 0.06. Similar to the mixing costs in the auditory modality, we observed a significant interaction between group and test, *F*_(2,33)_ = 3.84; *p* = 0.03; $\eta_{\text{G}}^{2}$ = 0.05, demonstrating that the auditory task-switching training in the AT group induced cross-modal transfer with reduced mixing costs in visual task switching, and in line with our previous work in younger individuals. Planned comparisons supported this conclusion, indicating that mixing costs decreased significantly in the AT group (pre: *M* = 726 ms; *SD* = 261 ms vs. post: *M* = 405 ms; *SD* = 159 ms), *t*_(11)_ = 4.61, *p* < 0.001, but not in the AC (pre: *M* = 618 ms; *SD* = 423 ms vs. post: *M* = 586 ms; *SD* = 318 ms), *t*_(11)_ = 0.27, *p* = 0.79, nor the PC group (pre: *M* = 739 ms; *SD* = 406 ms vs. post: *M* = 755 ms; *SD* = 370 ms), *t*_(11)_ = −0.19, *p* = 0.85.

### Cross-Modal Transfer: Visual Modality Switching Costs

Finally, we assessed the visual modality switching costs, which were also very small similar to those in the auditory modality (AT group pre: *M* = 25 ms; *SD* = 170 ms vs. post: *M* = −3 ms; *SD* = 93 ms; AC group pre: *M* = −34 ms; *SD* = 137 ms vs. post: *M* = 4 ms; *SD* = 138 ms; PC group pre: *M* = 8 ms; *SD* = 142 ms vs. post: *M* = −39 ms; *SD* = 150 ms). A two-way mixed ANOVA predictably failed to provide evidence in favor of any training related improvement or cross-modal transfer effects. There was no interaction between group and test, *F*_(2,33)_ = 0.46; *p* = 0.63; $\eta_{\text{G}}^{2}$ = 0.02, no main effect of group, *F*_(2,33)_ = 0.43; *p* = 0.65; $\eta_{\text{G}}^{2}$ < 0.01, and no main effect of test, *F*_(1,33)_ = 0.11; *p* = 0.74; $\eta_{\text{G}}^{2}$ < 0.01.

### Far Transfer: Number Stroop Task

The RTs on neutral (letters), compatible (digits corresponding to the number), and incompatible trials (digits not corresponding to the number) in the Number Stroop task at pre- and post-test are illustrated in Table 3. Training effects on the Number Stroop effect were tested with a 3 (group) × 2 (test) × 3 (trial type: neutral, compatible, incompatible) ANOVA with test and trial type as repeated measures factors. The analysis revealed a significant main effect of trial type, *F*_(2,66)_ = 20.80; *p* < 0.001; $\eta_{\text{G}}^{2}$ = 0.01, with longer RTs on incompatible trials (*M* = 904 ms; *SD* = 153 ms) than on neutral (*M* = 891 ms; *SD* = 153 ms) and compatible trials (*M* = 868 ms; *SD* = 166 ms), i.e., an overall compatibility effect in the Number Stroop task. There was also a significant main effect of test, *F*_(1,33)_ = 15.20; *p* < 0.001; $\eta_{\text{G}}^{2}$ = 0.07, with longer RTs at pre-test (*M* = 928 ms; *SD* = 165 ms) than at post-test (*M* = 848 ms; *SD* = 139 ms). However, the ANOVA revealed no significant interaction between test and trial type, *F*_(2,66)_ = 2.40; *p* = 0.10; $\eta_{\text{G}}^{2}$ < 0.01, suggesting that the Stroop effect did not change from pre- to post-test, and no three-way interaction, *F*_(4,66)_ = 0.67; *p* = 0.61; $\eta_{\text{G}}^{2}$ < 0.01, indicating that the auditory task-switching training did not exert an influence on the Stroop effect. All remaining effects were non-significant, *p*s > 0.10.

**Table 3:** Mean RTs (ms) in the Number Stroop task on neutral (letters), compatible (digits corresponding to the number of items) and incompatible trials (digits not corresponding to the number of items) in old adults.

**Trial type**	**Test**	**PC group**	**AC group**	**AT group**
Neutral	Pre	1,015 (132)	899 (213)	864 (73)
	Post	903 (124)	865 (154)	798 (124)
Compatible	Pre	985 (163)	892 (236)	850 (88)
	Post	848 (132)	847 (180)	784 (120)
Incompatible	Pre	1,036 (136)	913 (206)	894 (73)
	Post	903 (122)	870 (157)	809 (118)

*Standard deviations are shown in parentheses*.

### Far Transfer: Digit Span and Auditory Distraction

In the present study, the average number of correctly recalled items in the digit span task (i.e., number of digits recalled at the correct serial position) increased from pre-test (*M* = 4.06; *SD* = 1.57) to post-test (*M* = 4.49; *SD* = 1.43), *F*_(1,33)_ = 9.92; *p* = 0.003; $\eta_{\text{G}}^{2}$ = 0.03. Moreover, there was a significant main effect of the modality of the digits, *F*_(1,33)_ = 11.95; *p* = 0.001; $\eta_{\text{G}}^{2}$ = 0.04, with better recall of visually presented digits (*M* = 4.53; *SD* = 1.43) than for auditory digits (*M* = 4.06; *SD* = 1.57). However, there was no effect of the auditory task-switching training on digit span, as suggested by the absence of an interaction between group and test, *F*_(2,33)_ = 0.75; *p* = 0.48; $\eta_{\text{G}}^{2}$ < 0.01. In addition, there was no significant difference in digit span between the three groups, *F*_(2,33)_ = 2.59; *p* = 0.09; $\eta_{\text{G}}^{2}$ = 0.10, no interaction between group and modality, *F*_(2,33)_ = 1.29; *p* = 0.29; $\eta_{\text{G}}^{2}$ < 0.01, and no other significant effect, *F* < 1; *p* > 0.50.

As a measure of auditory distraction, Irrelevant Speech Effects (ISE) were calculated as the difference in the number of correctly recalled digits between trials with noise and speech as the irrelevant sound. The resulting ISE scores are illustrated in Table 4. A 3 (group) × 2 (test) mixed-factors ANOVA with test as a repeated-measures factor revealed no general change of the degree of auditory distraction from pre-test (*M* = 0.89; *SD* = 1.39 digits) to post-test (*M* = 0.95; *SD* = 1.06 digits), *F*_(1,33)_ = 0.11; *p* = 0.74; $\eta_{\text{G}}^{2}$ < 0.01. However, there was a marginally significant main effect of training group, *F*_(2,33)_ = 2.87; *p* = 0.07; $\eta_{\text{G}}^{2}$ = 0.09, with a considerably larger ISE in the PC group (*M* = 1.28; *SD* = 1.14) than in the two other groups (AC group: *M* = 0.70; *SD* = 1.20; AT group: *M* = 0.77; *SD* = 1.29). There was no interaction though, indicating that the auditory task-switching training did not reduce the degree of distraction produced by irrelevant speech, *F*_(2,33)_ = 0.11; *p* = 0.90; $\eta_{\text{G}}^{2}$ < 0.01.

**Table 4:** Irrelevant speech effects (digit span during speech subtracted from digit span during noise) for the visual and auditory serial recall task at pre- and post-test in old adults.

**Modality**	**Test**	**PC group**	**AC group**	**AT group**
Auditory	Pre	1.08 (1.04)	1.02 (1.28)	0.78 (1.58)
	Post	1.65 (0.70)	1.10 (1.17)	0.97 (1.18)
Visual	Pre	1.33 (1.73)	0.28 (1.27)	0.82 (1.39)
	Post	1.05 (0.85)	0.40 (0.96)	0.52 (1.10)

*Standard deviations are shown in parentheses*.

### Far Transfer: Corsi Span and Visual Distraction

Corsi span was calculated as the number of visual locations that were recalled in the correct serial position. In the present study, the number of correctly recalled square locations in the Corsi block task increased significantly from pre-test (*M* = 2.33; *SD* = 1.06) to post-test (*M* = 3.13; *SD* = 0.99), *F*_(1,33)_ = 40.82; *p* < 0.001; $\eta_{\text{G}}^{2}$ = 0.15, indicating a general improvement of visuo-spatial short-term memory in old adults. In addition, Corsi span was higher on trials without visual distractors (*M* = 2.82; *SD* = 1.16) than on trials with the visual distractors (*M* = 2.64; *SD* = 1.02), *F*_(1,33)_ = 11.98; *p* = 0.002; $\eta_{\text{G}}^{2}$ = 0.09, indicating general interference by irrelevant visual distractors. In Table 5, the degree of visual interference is illustrated separately for the pre- and post-tests in the three different groups. There was also a significant interaction between group and distractor, *F*_(2,33)_ = 5.13; *p* = 0.01; $\eta_{\text{G}}^{2}$ = 0.01, with more interference in the AT group than in the control groups (see Table 3). However, there was no interaction with test (pre, post), *F* < 1; *p* > 0.45, suggesting that the type of training did not affect the degree of visual distraction in the Corsi block task.

**Table 5:** Interference produced by visual distractors on the Corsi span (trials with visual distractors subtracted from trials without distractors), as measured at pre- and post-test in old adults.

**Test**	**PC group**	**AC group**	**AT group**
Pre	0.09 (0.39)	0.08 (0.59)	0.44 (0.58)
Post	0.11 (0.48)	−0.02 (0.48)	0.41 (0.54)

*Standard deviations are shown in parentheses*.

### Far Transfer: Fluid Intelligence

The percentage of correctly solved Ravens matrix problems differed significantly between three groups (see Table 6), *F*_(2,32)_ = 5.95; *p* = 0.006; $\eta_{\text{G}}^{2}$ = 0.21, but there was no interaction between group and test, *F*_(2,32)_ = 0.21; *p* = 81; $\eta_{\text{G}}^{2}$ < 0.01, suggesting that the task switching training did not affect fluid intelligence scores. There was also no main effect of test, *F*_(1,32)_ = 0.82; *p* = 0.37; $\eta_{\text{G}}^{2}$ < 0.01.

**Table 6:** Percentage of matrix problems solved by old adults at pre- and post-test.

**Test**	**PC group**	**AC group**	**AT group**
Pre	17.2 (10.1)	16.2 (13.5)	29.2 (15.9)
Post	17.7 (6.5)	19.9 (9.3)	30.6 (11.7)

*Standard deviations are shown in parentheses*.

### Age Comparisons of Training and Cross-Modal Transfer Effects on Task Switching Performance: Mixing and Switch Costs

In a final exploratory step, the reduced costs of task switching observed with old participants in the present study were contrasted with the analogous data set in young participants [as reported in Kattner et al. (2019)]. A four-factor cross-study ANOVA on the pre-post mixing costs, with test and modality (auditory, visual) as within subjects factors and age group (old, young) and training group as between-subjects factors revealed significant differences in mixing costs between the two age groups with larger mixing cost in the old adults (*M* = 609 ms; *SD* = 225 ms) than in the young adults (*M* = 422 ms; *SD* = 132 ms), *F*_(1,87)_ = 28.8; *p* < 0.001; $\eta_{\text{G}}^{2}$ = 0.14. While the training related decrease in mixing costs appeared more pronounced in the old adult cohort (compare Figures 2A,B), the three-way interaction between age group, training group and test was not significant, *F*_(2,87)_ = 2.59; *p* = 0.08; $\eta_{\text{G}}^{2}$ = 0.01. Since it is of theoretical interest whether this marginal aging difference was mediated by modality, as previous research has indicated potential for greater performance improvements in older compared with younger participants (Gaál and Czigler, 2018; Steyvers et al., 2019), a further exploratory follow-up analysis was conducted. However, neither three-way interaction was significant, either for auditory task switching, *F*_(2,87)_ = 2.62; *p* = 0.08; $\eta_{\text{G}}^{2}$ = 0.02, nor for visual task switching, *F*_(2,87)_ = 1.56; *p* = 0.28; $\eta_{\text{G}}^{2}$ < 0.01. Analysis of log transformed response times, which control for baseline performance differences due to age related slowing, converge with these results, suggesting no significant differences between age groups on the magnitude of mixing cost reductions in either modality.

For the switch costs (compare Figures 3A,B), the cross-study mixed-factors ANOVA revealed no significant difference between old and young individuals, *F*_(1,87)_ = 2.95; *p* = 0.09; $\eta_{\text{G}}^{2}$ < 0.01, no interaction between age group and test, *F*_(1,87)_ = 2.80; *p* = 0.07; $\eta_{\text{G}}^{2}$ < 0.01, as well as no other interactions with age group, *F* < 1; *p* > 0.36, indicating that the absence of a transfer of auditory task-switching training on the switch costs (in either modality) did not depend on the age of participants.

### Age Comparisons of Transfer Effects: Far Transfer

Although no substantial effects of auditory task-switching training on far transfer were observed in either this study with old participants, nor in Kattner et al. (2019), for completeness we report here the cross-study comparisons for each of the far transfer tests in a similar manner as described for the mixing and switching costs.

### Number Stroop

The cross-study analysis further revealed that old adults from the present study showed only significantly longer RTs in the Number Stroop task (*M* = 888 ms; *SD* = 138 ms) than the young adults from the previous study (*M* = 575 ms; *SD* = 81 ms; see Kattner et al., 2019), *F*_(1,86)_ = 202.50; *p* < 0.001; $\eta_{\text{G}}^{2}$ = 0.66.

### Digit Span and Auditory Distraction

The cross-study analysis further revealed a significant interaction between modality and age group, *F*_(1,87)_ = 12.24; *p* < 0.001; $\eta_{\text{G}}^{2}$ = 0.01, indicating that old adults in the present study recalled more visual digits than auditory digits, whereas there was even a small advantage for auditory items in the young adults of the previous study (auditory: *M* = 4.93; *SD* = 0.97; visual: *M* = 4.88; *SD* = 0.93). For the ISE scores, there was a significant interaction between test and age group, *F*_(1,87)_ = 7.13; *p* = 0.009; $\eta_{\text{G}}^{2}$ = 0.03, suggesting that the young participants of the previous study showed a more pronounced reduction of auditory distraction than the old adults of the present study (regardless of the type of training).

### Corsi Span and Visual Distraction

The cross-study analysis further revealed a significant interaction between age group and test, *F*_(1,87)_ = 7.07; *p* = 0.01; $\eta_{\text{G}}^{2}$ = 0.01, indicating that the pre-post improvement of Corsi span was more pronounced in the old adults of the present study (see above) than in the young adults of the previous study (from *M* = 4.25; *SD* = 1.00 to *M* = 4.65; *SD* = 0.79). For visual interference, there was only a marginally significant interaction between age group and training group, *F*_(1,87)_ = 2.46; *p* = 0.09; $\eta_{\text{G}}^{2}$ = 0.03, but no other effects.

### Fluid Intelligence

The cross-study ANOVA further revealed significant age differences in fluid intelligence scores, *F*_(1,86)_ = 280.16; *p* < 0.001; $\eta_{\text{G}}^{2}$ = 0.71 (old sample of present study: *M* = 21.9%; *SD* = 11.2% *vs*. young sample: *M* = 61.0%; *SD* = 11.9%), as well as a significant interaction between age group (study) and training group, *F*_(2,86)_ = 4.21; *p* = 0.02; $\eta_{\text{G}}^{2}$ = 0.07, but no interactions with test, suggesting that the type of training did not affect fluid intelligence in either age group.

## Discussion

In the present study, older participants (aged 60 and above) underwent four sessions of auditory task-switching training on a task which required them to flexibly shift auditory attention between two different task sets. Training resulted in considerable reductions in the task-switching mixing cost for stimuli in the trained auditory modality, as compared to non-improvement in two control groups. Additionally, the mixing cost reduction transferred to an untrained visual modality version of the task. Hence, this study provides several important findings. First is that task-switching training with an auditory modality selective attention task can specifically improve older individuals' cognitive flexibility as measured by reductions in the size of their task-switching mixing cost. Second, these effects can transfer across stimulus-modalities, which indicates that the capacity for amodal improvements to shifting is not limited to younger individuals (c.f. Kattner et al., 2019). Thirdly, we failed to find evidence to suggest these effects were larger in either age group. Finally, we failed to observe any far-transfer effects from the auditory task-switching training to four structurally dissimilar tasks measuring inhibitory control, working memory and fluid intelligence.

### Auditory Task Switching Training in Old Age

Previous task-switching training studies have predominately employed visual modality stimuli to train participants (Karbach and Kray, 2009; Zinke et al., 2012; Pereg et al., 2013; Kray and Fehér, 2017; Zhao et al., 2018), most likely due to the ease of presenting visual stimuli in a perceptually consistent manner. In contrast, the use of auditory modality tasks for task switching protocols, as opposed to mixtures of auditory and visual tasks (Strobach et al., 2012a), is uncommon (but see Koch et al., 2011; Lawo and Koch, 2014). Previous work by Koch et al. had focused their investigations of auditory attention shifting to the related concept of the cocktail party effect. They confirmed that shifting between different stimulus feature dimensions, such as ear and gender of auditory stimuli could produce switching costs. However, shifting between perceptual features of the spoken word stimuli, and shifting between different semantic categorizations represent different types of auditory attention shifting. In our previous study, Kattner et al. (2019) introduced the concept of auditory task set shifting, by probing whether younger adults could switch between a parity and a semantic categorization task presented in the auditory modality. Extending the findings of Koch et al. costs of switching were also observed, however these were predominately in the mixing cost rather than the switching cost. Furthermore, this study was the first to explicitly test whether shifting in the auditory modality could be improved with training. Additionally, Lawo and Koch (2014) reported an age-independent effect of auditory attention shifting in healthy aging. In this task, numeric categorizations of spoken number stimuli were made while shifting between perceptual characteristics of the relevant speaker. Although older individuals were slower to respond than younger ones, there was no age-difference in either error rates or switching costs.

Extending these lines of research, we have demonstrated for the first time that older participants can respond positively to cognitive training where auditory attention shifts were made between two orthogonal task sets, rather than just perceptual characteristics of auditory stimuli. Initially identifying that such switching induced costs in the older participant groups, we further observed that performance improved over the course of training. Specifically, response time and accuracy improved in both the active training (task-switching blocks) and active control (single-task blocks) groups. However, the reduction of the task-switching mixing cost following training was specific to the active training group only. This suggests that the type of training is an important factor in improving processes related to maintaining and coordinating multiple task-sets concurrently in working memory (Monsell, 2003), and confirms that the previously observed effects of training auditory attention shifting can be beneficial for older as well as younger age groups.

Interestingly, when we compared the mixing cost reduction for the older participants with our previously collected data from a younger cohort, we found a larger reduction for the older group. Although this effect was not statistically significant and appeared to be stronger in the trained auditory modality, this observation is not without precedent. Such age-dependent effects are a common observation in training protocols, where older subjects can sometimes attain similar performance as younger cohorts after training, despite having substantially larger costs at pre-test (Gaál and Czigler, 2018; Steyvers et al., 2019). This is indicative of the fact that cognitive flexibility itself may be a plastic resource, as proposed by Lövdén et al. (2010), and that some of the executive function limitations normally associated with aging may be more apparent than real; cognitive training protocols represent a key which may unlock this potential. Therefore, our data is in line with existing literature showing old adults can benefit more from task-switching training than young adults, at least for the mixing cost and its associated executive control processes. However, no transfer effects were observed for the switching costs. These were small, at around 50 ms and below in both the auditory and the visual modality (for comparison, switch costs exceeded 100 ms at both pre- and post-test for nearly all conditions in the study of Karbach and Kray, 2009). In some cases, the effect was actually a switching benefit, although this appears to be consistent across age groups. This will be briefly discussed in a later section.

### Cross-Modal Transfer Is Not Limited by Cognitive Aging

Cognitive decline across the age span has been attributed to reductions in processing speed (Salthouse, 1996, 2000) or cognitive flexibility (Lövdén et al., 2010), strategic biases (Starns and Ratcliff, 2010), or deficits in set-shifting ability (Ridderinkhof et al., 2002). Indeed, the task-switching mixing cost itself is known to co-vary with age (Wasylyshyn et al., 2011), and thus may also reflect deficits associated with cognitive aging. It could be suggested that some aspects of cognitive aging such as reductions in cognitive flexibility or perseverative behavior would constrain the capacity for cross-modal transfer effects in older adults. In the present study we provide evidence to the contrary; not only did mixing cost reductions transfer to the visual modality in older subjects who partook in auditory task-switching training, this reduction was of a similar magnitude to that previously observed in younger participants (Kattner et al., 2019). That is to say, we present evidence that cognitive aging is not a limiting factor for older subjects to improve set shifting mechanisms at an amodal processing level (at least for the current age range).

The modality-*in*dependent effects observed in this study and that of Kattner et al. (2019) can also be compared to other reports of modality-*dependent* transfer effects (e.g., Yeung et al., 2006; Karbach and Kray, 2009; Gaál and Czigler, 2018). In these studies, near-transfer is accounted for by the observation that different stimulus sets are used in training and testing, suggesting that it occurs when training leads to improvements in the general control mechanisms subtending set-shifting. However, since all stimuli were presented in a single (visual) modality, the question of whether this improvement iterated over *truly* general control mechanisms, or only those in the visual pathway, is outstanding. Together with Kattner et al. (2019), we provide reliable evidence that such near-transfer effects can occur at amodal control levels, thus extending our current understanding about the boundaries of transfer effects following task-switching training.

### On Small Switch Costs

Despite observing reliable mixing costs in accordance with our expectations, the magnitude of the switch costs was rather small. Instead of a phenomenological explanation, this may have been due to the use of an on-screen cue indicating the current trial, whose onset was the same as the task-stimuli. In alternating runs designs (Rogers and Monsell, 1995), the predictable structure of task-switch and repetition trials should be internalized by the subjects, encouraging anticipatory set-shifting, thus producing residual switch costs. However, our subjects may have only minimally engaged in anticipatory set-shifting, and chose to ignore the alternating runs structure in favor of waiting to see the concurrent trial-cue. Since we observed a similar pattern of effects in both age groups and modalities, it seems more likely that this unusual result was due to methodological than phenomenological reasons. Nonetheless, we observed no statistical difference in the magnitude of these switch costs across age groups, an effect which conforms to previous literature (Wasylyshyn et al., 2011).

### Absence of Far Transfer

Finally, we report an absence of far transfer effects. As mentioned in the introduction, far transfer effects following task-switching training are elusive, whereby a number of studies following the original Karbach and Kray (2009) paper which sought far transfer effects using a comparable study design, failed to find them (Pereg et al., 2013). Pereg et al. (2013) proposed that transfer must be dependent on the working memory demand of the trained tasks. However, Kray and Fehér (2017) systematically manipulated working memory and inhibition demands and failed to observe the hypothesized far transfer effects. Likewise, training dosage may be a critical factor in inducing far transfer in these designs, but the present study, and Kattner et al. (2019) and Zhao et al. (2018), trained participants with more trials than Karbach and Kray (2009) did, but failed to observe far-transfer effects. We conclude that, similar to Kattner et al. (2019), the auditory dichotic task-switching training transferred in a limited way to set-shifting executive functions, and did not appear to translate beyond this.

### Limitations and Future Directions

Two studies have now shown auditory-visual cross-modal transfer of mixing cost reductions following task-switching training. However, there are some caveats with the present study design that should be considered for future investigations. Firstly, training effects were confounded with sleep. A recent study has shown that generalization of statistical learning is possible from the auditory to the visual modality, but only after 24 h of sleep (Durrant et al., 2016). The degree of cross-modal transfer in that study was, in fact, predicted by the amount of slow-wave sleep experienced by participants. This raises the interesting possibility that cross-modal transfer following task-switching training might be limited to circumstances where sleep is permitted between the pre- and post-tests and sleep related consolidation of learning is a necessary prerequisite for the amodal processes to be trained. Carefully designed training protocols which control the amount of sleep between training sessions may be able to probe this moderating factor.

Additionally, in the present study the auditory and visual tasks were identical in both structure and the stimuli used. In other studies, near transfer effects are often observed when the stimuli and their categorization rules differ between the training and transfer tasks (Karbach and Kray, 2009; Zhao et al., 2018). It cannot yet be ruled out that the cross-modal task-switching effect was limited to the specific stimuli that were used, and may not transfer to untrained modality tasks with untrained stimulus- or task-sets. This should also be considered in further investigations of cross-modal transfer, by introducing stimulus sets and categorization rules in the testing phase which differ from that experienced by participants in the training phase.

Finally, we need to consider that the crossmodal training and transfer effect was observed in both this study and that of Kattner et al. (2019) only from the auditory to the visual modality. However, it has been observed that while older individuals are more susceptible than younger ones to distracting information in the visual modality when performing an auditory modality task, the same age difference might not relate to the reversed scenario (Guerreiro et al., 2013). Additionally, a heterogeneous decline of sensory systems with advancing age seems to contribute to older adults' susceptibility to multisensory illusions (Hirst et al., 2019), such that stronger auditory perception is used to compensate when visual acuity is weaker. Together these results seem to suggest a stronger reliance on visual than auditory information as age increases. This might result in a different pattern of cross-modal training and transfer effects if the trained task would have been presented in the visual modality. However, whether this would be the cause is open to further investigation and shows that it is necessary to test whether the crossmodal transfer effect pattern could be generalized to other sensory modalities.

## Conclusion

In this study, we addressed whether the possibility for cross-modal transfer effects following task-switching cognitive training is preserved in old age. We employed a complex design with one active training and two control groups to show that reductions in the mixing cost following training was specific to individuals who trained with task-switching. We also showed this reduction was present in an untrained visual-modality version of the task, of a comparable magnitude to that observed in a younger cohort. These findings suggest that near-transfer effects following task-switching reflect rather general improvements to relevant executive control functions at an amodal processing level. Moreover, despite the limitations associated with cognitive aging, the capacity for cross-modal transfer appears to be preserved in old age. Finally, we were unable to observe far transfer effects following training, although this seems to be representative of a growing body of evidence indicating that far transfer is rather elusive, at least following task-switching training.

## Data Availability Statement

The raw data supporting the conclusions of this article will be made available by the authors, without undue reservation.

## Ethics Statement

The studies involving human participants were reviewed and approved by Local committee approval at Technical University of Darmstadt. The patients/participants provided their written informed consent to participate in this study.

## Author Contributions

FK and TS conceived and designed the study. FK programmed the experiment. BT ran the experiment and collected the data. FK, BT, and TS analyzed, interpreted the data, and wrote the manuscript.

## Conflict of Interest

The authors declare that the research was conducted in the absence of any commercial or financial relationships that could be construed as a potential conflict of interest.
